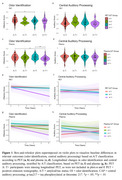# The role of tau and amyloid pathology in neurosensory impairments during preclinical Alzheimer's disease

**DOI:** 10.1002/alz70856_104086

**Published:** 2025-12-26

**Authors:** Daniel C Bowie, Yara Yakoub, Valentin Ourry, Ting Qiu, Fernando Gonzalez‐Ortiz, Kaj Blennow, Henrik Zetterberg, Judes Poirier, Sylvia Villeneuve, PREVENT‐AD Research Group

**Affiliations:** ^1^ McGill University, Montreal, QC, Canada; ^2^ Douglas Mental Health University Institute, Centre for Studies on the Prevention of Alzheimer's Disease (StoP‐AD), Montréal, QC, Canada; ^3^ Integrated Program in Neurosciences, McGill University, Montreal, QC, Canada; ^4^ Integrated Program in Neurosciences, McGill University, Montréal, QC, Canada; ^5^ Department of Psychiatry and Neurochemistry, Institute of Neuroscience and Physiology, The Sahlgrenska Academy at the University of Gothenburg, Gothenburg, Sweden; ^6^ Department of Psychiatry and Neurochemistry, Institute of Neuroscience and Physiology, The Sahlgrenska Academy, University of Gothenburg, Mölndal, Sweden; ^7^ Clinical Neurochemistry Laboratory, Sahlgrenska University Hospital, Mölndal, Sweden; ^8^ Clinical Neurochemistry Laboratory, Sahlgrenska University Hospital, Mölndal, Västra Götalands län, Sweden; ^9^ Department of Neurodegenerative Disease, UCL Institute of Neurology, London, United Kingdom; ^10^ Hong Kong Center for Neurodegenerative Diseases, Hong Kong, Hong Kong, China; ^11^ UW Department of Medicine, School of Medicine and Public Health, Madison, WI, USA; ^12^ Department of Psychiatry, Faculty of Medicine, McGill University, Montréal, QC, Canada; ^13^ McGill Centre for Integrative Neuroscience, McGill University, Montreal, QC, Canada; ^14^ Department of Psychiatry, McGill University, Montréal, QC, Canada

## Abstract

**Background:**

Prior cross‐sectional studies in the PREVENT‐AD cohort indicate that increased concentrations of *p*‐tau181 in cerebrospinal fluid are linked to impairments in odor identification (OI; Lafaille‐Magnan et al., 2017) and central auditory processing (CAP; Tuwaig et al., 2017). However, the extent to which AD pathology is predictive of change in neurosensory impairments remains unclear. To clarify these relationships, we examined how OI and CAP differ, cross‐sectionally and longitudinally, according to amyloid and tau (A/T) status based on PET. To support our findings, we replicated our results using baseline plasma A/T status.

**Method:**

We studied 233 participants from the PREVENT‐AD cohort who had at least one value for each of the following measures: amyloid PET ([^18^F]NAV4694), tau PET ([18F]AV‐1451), University of Pennsylvania Smell Identification Test (UPSIT), and Dichotic Sentence Identification test (DSI). Amyloid and tau PET thresholds were Centiloid=18 and SUVR=1.29, respectively. A partially overlapping subset of participants (*n* = 208) had values for plasma Aβ_42_ and Aβ_40_ concentrations measured using ultrasensitive immunoprecipitation coupled with mass spectrometry (IP‐MS) technique. Plasma *p*‐tau217 concentrations were quantified using the University of Gothenburg assay. Plasma amyloid positivity was determined by implementing a data‐driven algorithm (Dumurgier et al., 2022). Kruskal‐Wallis and Dunn's tests were conducted to assess baseline differences in olfaction and audition according to PET or plasma A/T status. Linear mixed effects models analyzed change over time in neurosensory abilities, by A/T group, while controlling for age, sex, and education.

**Results:**

For PET‐based classification, A+T− exhibited lower OI scores than A−T− (Figure 1a). For plasma‐based classification, A+T+ displayed lower OI scores than A+T− and A−T− (Figure 1c). Longitudinally, the rate of decline in OI was largest in A+T+, but this result was only significant for plasma (Figure 1g). No associations were found with CAP.

**Conclusion:**

Our analyses suggest that the presence of pathological amyloid and tau (A+T+) is associated with declines in the neurosensory domain of odor identification. Future studies should examine whether similar trends are observed for other neurosensory domains such as vision and gustation.